# Nitric Oxide Synthase Inhibition as a Neuroprotective Strategy Following Hypoxic–Ischemic Encephalopathy: Evidence From Animal Studies

**DOI:** 10.3389/fneur.2018.00258

**Published:** 2018-04-19

**Authors:** Laurent M. A. Favié, Arlette R. Cox, Agnes van den Hoogen, Cora H. A. Nijboer, Cacha M. P. C. D. Peeters-Scholte, Frank van Bel, Toine C. G. Egberts, Carin M. A. Rademaker, Floris Groenendaal

**Affiliations:** ^1^Department of Clinical Pharmacy, University Medical Center Utrecht, Utrecht, Netherlands; ^2^Department of Neonatology, Wilhelmina Children’s Hospital, University Medical Center Utrecht, Utrecht, Netherlands; ^3^Department of Pharmacy, Academic Medical Center, Amsterdam, Netherlands; ^4^Laboratory of NeuroImmunology and Developmental Origins of Disease (NIDOD), University Medical Center Utrecht, Utrecht, Netherlands; ^5^Department of Neurology, Leiden University Medical Center, Leiden, Netherlands; ^6^Brain Center Rudolf Magnus, University Medical Center Utrecht, Utrecht, Netherlands; ^7^Department of Pharmacoepidemiology and Clinical Pharmacology, Faculty of Science, Utrecht University, Utrecht, Netherlands

**Keywords:** nitric oxide synthase inhibition, neuroprotection, animal models, hypoxic–ischemic encephalopathy, 2-iminobiotin, review

## Abstract

**Background:**

Hypoxic–ischemic encephalopathy following perinatal asphyxia is a leading cause of neonatal death and disability worldwide. Treatment with therapeutic hypothermia reduced adverse outcomes from 60 to 45%. Additional strategies are urgently needed to further improve the outcome for these neonates. Inhibition of nitric oxide synthase (NOS) is a potential neuroprotective target. This article reviews the evidence of neuroprotection by nitric oxide (NO) synthesis inhibition in animal models.

**Methods:**

Literature search using the EMBASE, Medline, Cochrane, and PubMed databases. Studies comparing NOS inhibition to placebo, with neuroprotective outcome measures, in relevant animal models were included. Methodologic quality of the included studies was assessed.

**Results:**

26 studies were included using non-selective or selective NOS inhibition in rat, piglet, sheep, or rabbit animal models. A large variety in outcome measures was reported. Outcome measures were grouped as histological, biological, or neurobehavioral. Both non-selective and selective inhibitors show neuroprotective properties in one or more outcome measures. Methodologic quality was either low or moderate for all studies.

**Conclusion:**

Inhibition of NO synthesis is a promising strategy for additional neuroprotection. In humans, intervention can only take place after the onset of the hypoxic–ischemic event. Therefore, combined inhibition of neuronal and inducible NOS seems the most likely candidate for human clinical trials. Future studies should determine its safety and effectiveness in neonates, as well as a potential sex-specific neuroprotective effect. Researchers should strive to improve methodologic quality of animal intervention studies by using a systematic approach in conducting and reporting of these studies.

## Introduction

Hypoxic–ischemic encephalopathy (HIE) following perinatal asphyxia (i.e., severe oxygen deprivation at birth) is one of the leading causes of neonatal death and adverse neuromotor outcome in term and near-term infants worldwide. In high-income countries, the incidence of HIE has been estimated between 0.5 and 1.0 for every thousand live births, although some sources have reported an incidence as high as 8 per 1,000 live births (1, 2). In low- and middle-income countries, the incidence of HIE is higher, affecting more than 1.1 million babies annually (3–5).

The overall burden of HIE is high, in terms of quality-adjusted life years, years of life lost, and years lived with disability, not to mention a great financial cost for both society and the families involved (6, 7). With an estimated annual one million deaths worldwide, HIE is accountable for roughly 25% of all deaths in the neonatal period (3, 8).

Hypoxic–ischemic brain injury is not a single event, evoked by the actual asphyxia, but rather an ongoing process that leads to significant neuronal cell death over hours to days after the initial insult (9, 10). Several distinct phases have been identified in this process. The primary energy failure takes place during the hypoxic–ischemic event, resulting in failure of oxidative metabolism, cytotoxic edema, and accumulation of excitotoxins (11). After resuscitation and restoration of cerebral circulation, a latent phase, lasting approximately 6 h, commences (12, 13). Subsequently, starting between 6 and 15 h after asphyxia, the brain experiences a secondary energy failure that can last for days. This phase is marked by seizures, renewed cytotoxic edema, release of excitotoxins, impaired cerebral oxidative energy metabolism, and finally, neuronal cell death (14).

Currently, the only treatment that has proven to effectively reduce hypoxic–ischemic brain injury following perinatal asphyxia is the application of therapeutic hypothermia (TH). During TH the brain temperature is lowered to 33–34°C which is maintained for 72 h (1). Since the introduction of TH, the combined adverse outcome of death and disability, such as hearing loss, cerebral palsy, and other neuromotor disorders, has been reduced from approximately 60–45% (15–17). TH has widely been implemented as the standard of care treatment for moderate to severe HIE in high-income countries. However, TH needs to be started within 6 h after birth, leaving clinicians with a narrow window for establishing the diagnosis and severity of HIE as well as transportation to a medical facility equipped for TH (18). Additional neuroprotective strategies for HIE are urgently needed to augment TH, but when hypothermia is not yet feasible, act as a first line treatment option (3, 4, 19).

A potential target for (additional) neuroprotection in patients with HIE is the inhibition of nitric oxide synthase (NOS, enzyme commission number 1.14.13.39). NOS is an enzyme catalyzing production of nitric oxide (NO) from l-arginine. After perinatal asphyxia, NO can react with the superoxide free radical to form toxic peroxynitrite, setting a pre-apoptotic pathway in motion, resulting in neuronal loss (10, 20). Nitrotyrosine, an end product of this process, has been demonstrated post mortem in neonatal brain and spinal cord tissue after severe HIE (21, 22).

Three isoforms of NOS have been identified: endothelial (eNOS), neuronal (nNOS), and inducible NOS (iNOS) (23). All isoforms are upregulated after asphyxia; both nNOS and eNOS immediately after reperfusion and iNOS from several hours onward (24). While eNOS is regarded to be critical in maintaining pulmonary blood flow, preventing pulmonary hypertension and thereby maintaining adequate oxygenation of tissues throughout the body, excessive activation of nNOS and iNOS is associated with deleterious effects on the brain (24, 25). To illustrate, in mice genetically deficient of eNOS, infarct size after middle cerebral artery occlusion is larger compared with wild-type animals, due to a reduction in regional cerebral blood flow (26). By contrast, nNOS knockout mice are protected against hypoxic–ischemic brain injury, while mice lacking iNOS showed a delayed reduction in brain injury (27–32).

The aim of this study is to review the available evidence on NOS inhibition as a potential neuroprotective strategy in animal models translational for neonatal HIE and to identify one or more NOS inhibiting compounds that could evolve from preclinical to clinical studies in the near future.

## Methods

### Search Strategy

Studies assessing the neuroprotective effects of NOS inhibitors in HIE models were identified. A literature search using the EMBASE, Medline, Cochrane, and PubMed databases was performed. The primary keywords were *Animals (newborn), Hypoxia*, and *Nitric Oxide Synthesis*; the searches were limited to the English language. The complete search string is included in Supplementary Material. After the exclusion of duplicates, the titles and abstracts were independently screened by two researchers (Laurent M. A. Favié and Arlette R. Cox). A final selection was made after full text evaluation. Any discrepancies were resolved by a third researcher (Floris Groenendaal). In addition, the reference lists of the retrieved articles were searched for additional studies.

### Selection Criteria

Studies were included based on the following inclusion criteria: animal models of a postnatal age in which brain development corresponds to near term or term brain development in humans, transient hypoxia or hypoxia–ischemia (HI), neuroprotection as outcome defined by histological, biochemical, and/or neurobehavioral parameters and inclusion of both a treatment group administering at least one NOS inhibitor and a control group that received sham treatment or consisted of untreated animals.

### Data Synthesis

The year of publication, name of first author, the class and type of NOS inhibitor, the animal model, the method used to achieve HI, the dose and number of animals in each treatment group, the type of control group and number of control animals, the timing of administration with regards to the HI insult, and the results on the reported outcome parameters were recorded for each study. Each outcome parameter was categorized as histological, biochemical, or neurobehavioral.

### Quality Assessment

The methodological quality of the included articles was assessed using the SYRCLE’s risk of bias (RoB) tool for animal intervention studies (33). This tool is based on the Cochrane RoB tool and consists of 10 items on which an article can be scored. Each item was scored 0, 1, or 2 points by two researchers (Laurent M. A. Favié and Arlette R. Cox) independently. If no evidence for adherence or evidence for non-adherence was found, a score of 0 was awarded. When evidence for adherence was present but inconclusive, one point was scored. If the item was fully adhered to, two points were scored. Any discrepancies were resolved after consultation with a third researcher (Agnes van den Hoogen). Because of the nature of the included studies and the timing of the interventions, “allocation concealment” was deemed unfeasible and was not rated for any of the articles. Articles scoring 1–6 points were considered low quality, 7–12 points moderate quality, and 13–18 points high quality. An example of the tool is included in Supplementary Material.

## Results

### Eligible Studies

**Figure 1:**
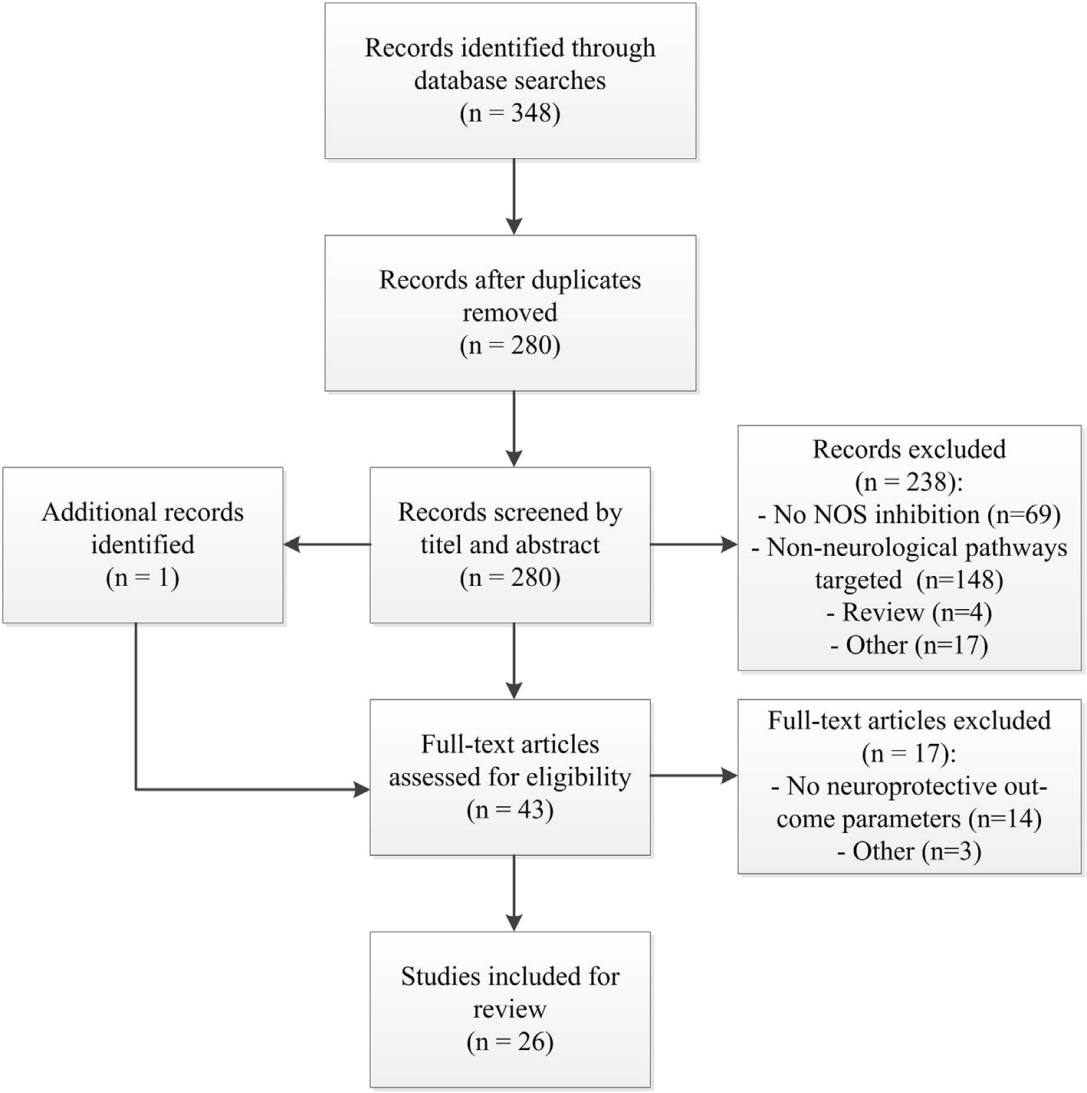
Study flow diagram. Abbreviations; *n*, number of studies; NOS, nitric oxide synthase.

The search yielded a total of 348 studies; 280 studies after removal of duplicates. After screening of title and abstract, 238 articles were excluded. Screening of the reference lists identified one additional article. 43 articles were thus assessed in full detail. Of these, 26 were deemed eligible for inclusion (Figure 1); the data were extracted from these studies, and these studies were assessed for methodological quality. Performing a meta-analysis was considered impossible because of the heterogeneity of the studies in outcome, administered NOS inhibitor, and animal models.

### Study Characteristics

The included studies and their descriptive characteristics are summarized in Table 1. Eight studies (31%) tested a non-specific NOS inhibitor (34–41), another eight (31%) applied an nNOS-specific inhibitor (42–49); three studies (12%) used an iNOS-specific inhibitor (50–52); and six (23%) used an inhibitor of both nNOS and iNOS (53–58). One study (3%) used separate groups for nNOS and iNOS inhibition (59). Four different species of animals were used: rat (*n* = 11, 42%), piglet (*n* = 10, 38%), sheep (*n* = 3, 12%), and rabbit (*n* = 2, 8%).

**Table 1:** Study characteristics including RoB score.

First author (year)	NOS inhibitor class, type	Animal, age	HI method	Dose, no., RoA	Control, no., RoA	Timing	Outcome		RoB L/M/H (score)
							Parameter H/B/N, NP yes/no	Result	

Trifiletti (1992) (34)	Non-spec, NNLA	Rat (Sprague-Dawley), 7 days	Left carotid artery ligation and hypoxia (FiO_2_ 0.08) for unknown duration	50 mg/kg, *n* = 6, ip	Vehicle, *n* = 6, ip	15 h before insult	H, yes	89% reduction in ipsilateral/contralateral weight ratio disparity vs vehicle	L (3)
				100 mg/kg, *n* = 4, ip			H, yes	100% reduction in ipsilateral/contralateral weight ratio disparity vs vehicle	

Hamada (1994) (35)	Non-spec, NNLA	Rat (Wistar), 7 days	Left carotid artery ligation and hypoxia (FiO_2_ 0.08) for 150 min	2 mg/kg, *n* = 12, ip	Vehicle, *n* = 12, ip	90 min before insult	H, yes	Reduction in cortical and striatal lesions vs vehicle	M (7)
				2 mg/kg, *n* = 12, ip	Vehicle, *n* = 12, ip	Directly after insult	H, no	No reduction in cortical and striatal lesions vs vehicle	

Nunagami (36)	Non-spec, NNLA	Piglet (unknown), 2–4 days	Hypoxia (FiO_2_ 0.07) for 60 min	40 mg/kg, *n* = 5, iv	Vehicle, *n* = 5, iv	60 min before insult	B, yes	Significant decrease in free radical formation of 65% vs vehicle Preservation of Na–K–ATPase activity vs vehicle Significant reduction in lipid peroxidation vs vehicle	M (7)

Groenendaal (1999) (37)	Non-spec, NNLA	Piglet (Yorkshire), 1–3 days	Bilateral carotid artery occlusion and hypoxia (FiO_2_ 0.07) for 60 min	40 mg/kg, *n* = 5, iv	Vehicle, *n* = 5, iv	60 min before insult	B, no	Worse cerebral energy status during and after HI vs vehicle (no change before HI)	L (6)

Ashraf (2002) (38)	Non-spec, NNLA	Piglet (unknown), 3–5 days	Hypoxia (FiO_2_ 0.05–0.15) for 60 min	40 mg/kg, *n* = 6, NA	Untreated, *n* = 9, NA	Unknown time before insult	B, yes	Prevention of hypoxia-induced upregulation of nitrated Bax protein vs untreated	L (5)

Zubrow (2002) (39)	Non-spec, NNLA	Piglet (Yorkshire), 2–4 days	Hypoxia (FiO_2_ 0.07–0.09) for 60 min	40 mg/kg, *n* = 7, iv	Vehicle, *n* = 6, iv	60 min before insult	B, yes	Significant decrease in amount of Bax protein and DNA fragmentation vs vehicle	L (6)

Dorrepaal (1997) (40)	Non-spec, NNLA	Sheep (Romney/Suffolk), 2–11 days	Hypoxia (FiO_2_ 0.06–0.08) for 30 min followed by MABP < 35 mmHG for 5 min	10 mg/kg, *n* = 6, iv	Vehicle, *n* = 6, iv	Directly after insult	H, yes	Non-significant lower brain–body mass ratio vs vehicle Non-significant decrease in necrotic Purkinje cells vs vehicle	M (8)
							B, yes	Significant increase in cerebral metabolic oxygen rate vs vehicle Significant recovery of electrocortical brain activity to baseline vs vehicle	
				40 mg/kg, *n* = 6, iv			H, yes	Significant lower brain–body mass ratio vs vehicle Non-significant decrease in necrotic Purkinje cells vs vehicle	
							B, yes	Significant increase in cerebral metabolic oxygen rate vs vehicle	
							B, no	No change in recovery of electrocortical brain activity to baseline vs vehicle	

Blumberg (1991) (41)	Non-spec, l-NAME	Rat (Wistar), 14 days	Right common artery ligation and hypoxia (FiO_2_ 0.08) for 90 min	30 mg/kg, *n* = 16, ip	Vehicle, *n* = 18, ip	Directly after insult	H, no	No significant difference in size of infarction vs vehicle	M (8)

Ishida (2001) (42)	nNOS, 7-NI	Rat (CD), 7 days	Right common artery ligation and hypoxia (FiO_2_ 0.08) for 120 min	50 mg/kg, *n* = NS, ip	Vehicle, *n* = NS, ip	30 min before insult	H, no	No neuroprotection vs vehicle	L (5)
				100 mg/kg, *n* = NS, ip		30 min before insult	H, yes	Significant reduction in the difference between the ipsilateral and contralateral cerebral hemisphere wet weights vs vehicle	
				50 mg/kg, *n* = NS, ip		15 min after insult	H, no	No neuroprotection vs vehicle	
				100 mg/kg, *n* = NS, ip		15 min after insult	H, no	No neuroprotection vs vehicle	

Parikh (2003) (43)	nNOS, 7-NI	Piglet (unknown), 3–5 days	Hypoxia (FiO_2_ 0.05–0.07) for 60 min	1 mg/kg, *n* = 6, ip	Untreated, *n* = 5, ip	Directly before insult	B, yes	Less caspase-3 activity and less DNA fragmentation vs untreated	L (6)

Ashraf (2004) (44)	nNOS, 7-NI	Piglet (Yorkshire), 2–4 days	Hypoxia (FiO_2_ 0.05–0.15) for 60 min	NA, *n* = 6, NA	Untreated, *n* = 5, NA	Unknown time before insult	B, yes	Prevention of hypoxia-induced decrease in protein tyrosine phosphatases activity vs untreated	L (5)

Yu (2011) (45)	nNOS, 7-NI	Rabbit (New-Zealand White), embryonic day 22 (70% gestation)	Uterine ischemia for 40 min	0.1575 μmol/kg, *n* = NS, iv	Vehicle, *n* = NS, iv	30 min before insult	N, yes	Decrease in number of deaths vs vehicle Significantly improved righting reflex vs vehicle	M (7)
	nNOS, JI-8			0.1575 μmol/kg, *n* = NS, iv			N, yes	Significant increase in normal appearing kits vs vehicle; significant decrease in severely affected and dead kits vs vehicle Significantly improved smell, muscle tone, and righting reflex vs vehicleOverall better outcome vs 7-NI	

Mishra (2006) (46)	nNOS, JI-10	Piglet (Yorkshire), 3–5 days	Hypoxia (FiO_2_ 0.06) for 60 min	1 mg/kg, *n* = 5, ip	Untreated, *n* = 5, ip	Directly after insult	B, yes	Decreased expression of Bax protein and DNA fragmentation vs untreated	L (6)

Drury (2013) (47)	nNOS, JI-10	Sheep (Romney/Suffolk), GA 103–104 (term = 147 days)	Complete umbilical cord occlusion for 25 min	0.044, *n* = 8, iv	Vehicle, *n* = 8, iv	15 min before insult	H, yes	Partial neuronal and white matter protection after 7 days recovery vs vehicle	M (10)
							B, yes	Delay in the onset of seizures on EEG vs vehicle	

Drury (2014) (48)	nNOS, JI-10	Sheep (Romney/Suffolk), GA 103–104 (term = 147 days)	Complete umbilical cord occlusion for 25 min	0.022 mg/kg, *n* = 8, iv	Vehicle, *n* = 8, iv	30 min before insult	H, yes	Significant reduction in loss of striatal phenotypic neurons vs vehicle	M (10)

Ji (2009) (49)	nNOS, C5 or C6	Rabbit (New-Zealand White), embryonic day 22 (70% gestation)	Uterine ischemia for 40 min	NA, *n* = NS, iv	Vehicle, *n* = NS, iv	30 min before insult	N, yes	Less fetal/neonatal deaths vs vehicle Less neurobehavioral abnormalities vs vehicle More normal kits at P1 vs vehicle	L (5)
	nNOS, C6			NA, *n* = NS, iv		30 min after insult	N, no	No difference in fetal/neonatal deaths vs vehicle No difference in neurobehavioral abnormalities vs vehicle No difference in normal kits at P1 vs vehicle	

Ikeno (2000) (50)	iNOS, S-MI	Rat (Wistar), 7 days	Right carotid artery ligation and hypoxia (FiO_2_ 0.08) for 90 min	10 mg/kg, *n* = NS, ip	Vehicle, *n* = NS, ip	Directly before insult, repeated at 12, 24, 36, and 48 h	H, yes	Significantly decreased damage to the cerebral cortex vs vehicle	L (5)

Tsuji (2000) (51)	iNOS, AG	Rat (Wistar), 7 days	Left carotid artery ligation and hypoxia (FiO_2_ 0.08) for 150 min	300 mg/kg, *n* = 29, ip	Vehicle, *n* = 24, ip	60 min before insult, repeated every 8 h, nine doses in total	H, yes	Significant reduction in cortical infarct volume of 89% vs vehicle Significant reduction in striatal infarct volume of 90% vs vehicle	M (8)

Tutak (2005) (52)	iNOS, AG	Rat (Wistar), 7 days	Left carotid artery ligation and hypoxia for 150 min	300 mg/kg, *n* = 18, ip	Vehicle, *n* = 18, 30 min after insult, repeated every 12 h, ip	30 min after insult, repeated every 12 h, six doses in total	H, no	No reduction in mean infarcted area vs vehicle	M (12)
	iNOS, IMC			0.2 mg/kg, *n* = 20, ip		30 min after insult, repeated every 8 h, nine doses in total	H, no	No reduction in mean infarcted area vs vehicle	
	iNOS, AG and IMC			300 and 0.2 mg/kg, *n* = 18, ip		AG: 60 m before insult; IMC: 30 m after insult, repeated every 8 h, nine doses in total	H, yes	Significant reduction in mean infarcted area vs vehicle	

van den Tweel (2005) (53)	nNOS and iNOS, 2-IB	Rat (Wistar), 12 days	Right carotid artery ligation and hypoxia (FiO_2_ 0.08) for 90 min	5.5 mg/kg, *n* = 11, sc	Vehicle, *n* = 24, sc	Directly after insult, repeated at 12 and 24 h	H, no	No difference in hippocampus and cortex neuropathology score vs vehicle No difference in ipsilateral/contralateral hemisphere area ratio vs vehicle	M (8)
				10 mg/kg, *n* = 10, sc					
							H, yes	Significantly higher hippocampus and cortex neuropathology score vs vehicle	
				30 mg/kg, *n* = 20, sc					
							H, no	No difference in ipsilateral/contralateral hemisphere area ratio vs vehicle	
							B, yes	Significantly higher hippocampus and cortex neuropathology score vs vehicle Significantly higher ipsilateral/contralateral hemisphere area ratio vs vehicle
							B, yes	Significantly lower ipsilateral HSP70 level vs vehicle
							H, no	No difference in nitrotyrosine levels vs vehicle

Nijboer (2007) (54)	nNOS and iNOS, 2-IB	Rat (Wistar), 7 days	Right carotid artery ligation and hypoxia (FiO_2_ 0.08) for 120 min	10 mg/kg, *n* = NS, sc	Vehicle, *n* = NS, sc	Directly after insult, repeated at 12 and 24 h	H, yes	Significantly higher ipsilateral/contralateral hippocampus area ratio vs vehicle in females only Significant reduction in cortical and hippocampal lesions vs vehicle in females only	M (11)
							B, yes	Significant reduction in cytochrome c release vs vehicle in females only Decrease in caspase-3 activity vs vehicle in females only No effect on nuclear translocation of apoptosis-inducing factor vs vehicle in both genders	
							N, yes	Less deaths in female pups compared with male pups	

Peeters-Scholte (2002) (55)	nNOS and iNOS, 2-IB	Piglet (Dutch Store) 1–3 days	Bilateral carotid artery occlusion and hypoxia for 60 min	0.2 mg/kg, *n* = 11, iv	Vehicle, *n* = 12, iv	Directly after insult, repeated every 60 min, six doses in total	H, yes	90% reduction of vascular edema vs vehicle 60–80% increase in normal neuronal cells vs vehicle	M (7)
							B, yes	90% improvement of cerebral energy state vs vehicle Reduction of caspase-3 activity by 93% in cortex and 71% in striatum vs vehicle	

Peeters-Scholte (2002) (56)	nNOS and iNOS, 2-IB	Piglet (Dutch Store) 1–3 days	Bilateral carotid artery occlusion and hypoxia for 60 min	0.2 mg/kg, *n* = 11, iv	Vehicle, *n* = 12, iv	Directly after insult, repeated every 60 min, six doses in total	B, yes	Preservation of endogenous IGF-1 production vs vehicle Reduction of caspase-3 activity vs vehicle	L (4)
							B, no	No significant decrease in cytokine production vs vehicle	

Bjorkman (2013) (57)	nNOS and iNOS, 2-IB	Piglet (Yorkshire), newborn	Hypoxia (FiO_2_ 0.02–0.04) for 30 min	0.1 mg/kg, *n* = 7, iv	Vehicle, *n* = 10, iv	Directly after insult, repeated every 60 min, six doses in total	H, yes	Decreased nitration in thalamus, parietal and temporal cortex vs vehicle	M (11)
							H, no	No difference in neuronal injury histology score	
							B, no	No difference in electrographical seizure activity at 48 h vs vehicleNo difference in caspase-3 activity vs vehicle	
							N, yes	Increased survival with normal EEG at 48 h vs vehicle	
							N, no	No difference in neurobehavioral scores at 48 h vs vehicle	
				0.2 mg/kg, *n* = 9, iv			H, yes	Decreased nitration in thalamus, parietal and temporal cortex vs vehicle	
							H, no	No difference in neuronal injury histology score	
							B, yes	Lower electrographical seizure activity at 48 h vs vehicle	
							B, no	No difference in Caspase-3 activity vs vehicle	
							N, yes	Increased survival with normal EEG at 48 h vs vehicle	
							N, no	No difference in neurobehavioral scores at 48 h vs vehicle	
				1.0 mg/kg, *n* = 5, iv			H, yes	Decreased nitration in thalamus, parietal and temporal cortex vs vehicle	
							H, no	No difference in neuronal injury histology score	
							B, yes	Lower electrographical seizure activity at 48 h vs vehicle	
							B, no	No difference in caspase-3 activity vs vehicle	
							N, yes	Increased survival with normal EEG at 48 h vs vehicle	
							N, no	No difference in neurobehavioral scores at 48 h vs vehicle	

van den Tweel (2002) (58)	nNOS and iNOS, 7-NI and AG	Rat (Sprague-Dawley), 12 days	Right carotid artery ligation and hypoxia (FiO_2_ 0.08) for 90 min	50 and 100 mg/kg, *n* = 24, ip	Vehicle, *n* = 24, ip	Directly after insult, AG repeated every 12 h, four doses in total	H, yes	Significant reduction in brain damage to the ipsilateral hemisphere vs vehicle	M (12)
							B, no	No difference in HSP70 or cytokine mRNA expression vs vehicle	

Hsu (2014) (59)	nNOS, 7-NI	Rat (Sprague-Dawley), 7 days	Right carotid artery ligation and hypoxia (FiO_2_ 0.08) for 120 min	75 mg/kg, *n* = NS, ip	Vehicle, *n* = NS, ip	30 min before insult	H, yes	Higher ipsilateral/contralateral cortical area ratio vs vehicle and AG Significant increases in vascular density and decreases of BBB damage and microglia activation vs vehicle Decrease in microvascular nitrosative stress vs vehicle and AG	M (7)
							B, yes	Increased cerebral perfusion vs vehicle and AG	
						3 h after insult	H, no	No difference in ipsilateral/contralateral cortical area ratio vs vehicle	
	iNOS, AG			300 mg/kg, *n* = NS, ip		30 min before insult	H, yes	Higher ipsilateral/contralateral cortical area ratio vs vehicle Significant increases in vascular density and decreases of BBB damage and microglia activation vs vehicle	
							H, no	No change in microvascular nitrosative stress vs vehicle	
							B, no	No change in cerebral perfusion vs vehicle	
						3 h after insult	H, yes	Higher ipsilateral/contralateral cortical area ratio vs vehicle and 7-NI	

*Studies are grouped by class and type of NOS inhibitor and subsequently by type of animal tested and year of publication. Low quality studies are indicated by a gray background*.

*non-spec, non-specific; NNLA, N-nitro-l-arginine; l-NAME, N-nitro-l-arginine methyl ester; nNOS, neuronal nitric oxide synthase; 7-NI, 7-nitro indazole; iNOS, inducible nitric oxide synthase; S-MI, S-methyl-isothiourea; AG, aminoguanidine; IMC, indomethacin; 2-IB, 2-iminobiotin; GA, gestational age; FiO_2_, fraction of inspired oxygen; MABP, mean arterial blood pressure; RoA, route of administration; ip, intraperitoneal; iv, intravenous; sc, subcutaneous; NP, neuroprotection; H, histological; B, biochemical; N, neurobehavioral; HI, hypoxia–ischemia; HSP70, heat shock protein 70; EEG, electro-encephalogram; BBB, blood–brain barrier; RoB, risk of bias; L, low; M, moderate; NA, not available; NOS, nitric oxide synthase; NS, not specified*.

Different models for HI were used, mostly dependent on the animal species. All rat studies applied the Vannucci–Rice model in P7–P14 pups. All newborn (P1–P5) piglet studies induced brain injury by hypoxia for 30–60 min, in 30% of studies combined with transient bilateral artery occlusion. In sheep aged 2–11 days (one study), hypoxia for 30 min was combined with hypotension for 5 min. Also, two studies using sheep at 103–104 days gestation (term = 147 days) were included, in which brain injury was induced by hypoxia due to occlusion of the umbilical cord for 25 min. In rabbits, fetuses (embryonic day 22, 70% gestation) were subjected to an HI event by uterine ischemia for 40 min.

The dosing regimen of the included studies is summarized in Table 2. Seventeen studies (65%, all non-specific or nNOS-specific inhibitors) describe only a single administration, and nine studies (35%, all iNOS of combined nNOS and iNOS inhibitors) described repeated dosing. With regards to timing of the intervention, 12 studies (46%) administered the (first) dose before the onset of the HI event; 9 (35%) after the event; and the remaining 5 (19%) incorporated groups with administration both before and after the event.

**Table 2:** Dosing frequency and timing of intervention for the included studies.

Timing of first dose to HI event	Dosing frequency	Type of inhibitor				Total no of studies	
		Non-specific	nNOS	iNOS	nNOS + iNOS		
Prior	Single	5	5	–	–	10	12
	Repeated	–	–	2	–	2	
Post	Single	2	1	–	–	3	9
	Repeated	–	–	–	6	6	
Both	Single	1	3^a^	1^a^	–	4^a^	5^a^
	Repeated	–	–	1	–	1	

Total		8	9	4	6	26	

*^a^One study tested both an nNOS and iNOS inhibitior in separate groups*.

*non-spec, non-specific; nNOS, neuronal nitric oxide synthase; iNOS, inducible nitric oxide synthase; HI, hypoxia–ischemia*.

### Outcome

The results of the reported outcome parameters for each study are presented in Table 1. A wide variety of histological, biochemical, and neurobehavioral outcome parameters were reported. Histological parameters included ipsilateral/contralateral weight ratio disparity and analysis of cortical and striatal lesions. Biochemical parameters included free radical formation and other biomarkers for neurological damage, but also cerebral energy status and electrocortical brain activity. Neurobehavioral parameters included overall survival, survival with normal EEG and results of neurobehavioral tests.

In the group of non-specific NOS inhibitors, administration before onset of the insult proved neuroprotective in 7/8 settings (88%), while administration directly after the insult was partially beneficial in 2/3 settings (67%).

For nNOS inhibitors, administration before the insult showed neuroprotective properties in 9/10 settings (90%) and when administered directly after the insult (1/1). When administration was delayed by 15 min or more, neuroprotective properties were lost (4/4).

When treatment with an iNOS inhibitor was started before the insult, neuroprotection was achieved (4/4). Administering the first dose after induction of HI showed neuroprotection in 33% of the settings (1/3). Hsu et al. administered the iNOS inhibitor aminoguanidine (AG) 30 min before and 3 h after the insult as a single dose. Both were neuroprotective compared with the control group, although less parameters were tested in the post insult treatment group.

All studies testing combined inhibition of nNOS and iNOS reported (partially) neuroprotective outcome. van den Tweel et al. (53) showed that 2-iminobiotin (2-IB) is neuroprotective in rats in a dose-dependent matter.

A direct comparison between two different inhibitors was made in two studies. Yu et al. reported superior neuroprotection of the novel nNOS inhibitor JI-8 compared with 7-nitro indazole (7-NI) when administered before the insult in equimolar doses. Hsu et al. observed that both 7-NI and AG are neuroprotective when administered 30 min before HI and that 7-NI is superior to AG in this setting. When the compounds were administered 3 h after HI, 7-NI lost its neuroprotective effect while AG remained neuroprotective compared with both vehicle and 7-NI.

### Methodological Quality

Eleven studies (42%) were ranked low quality, 15 (58%) were considered moderate quality; none of the studies were ranked in the high quality group. On average, RoB score was 7 (3–12). Overall, animal baseline characteristics, randomization for treatment allocation, blinding of investigators and/or outcome assessors, and random selection for outcome assessment were often not mentioned and therefore scored 0.

## Discussion

This systematic review shows that both selective and non-selective NOS inhibitors have neuroprotective qualities in various animal models of HI brain damage using histological, biochemical, and neurobehavioral outcome parameters. In animal studies, induction of the insult and administration of the potentially neuroprotective agent (before and/or after the insult) can be timed precisely. By contrast, this is not the case in clinical practice. The onset of perinatal asphyxia is often sudden and unpredictable. Therefore, administration of any drug before the onset of the insult is impossible, and administration directly after the insult (i.e., directly after birth) is highly improbable. All non-selective NOS inhibitors reviewed in this study were administered before insult or directly after; there are no data on delayed administration. Furthermore, non-selective inhibitors will also target eNOS, which could counteract the potential neuroprotective effects of nNOS and iNOS inhibition. For selective nNOS inhibitors, neuroprotection was lost when administration was withheld by as little as 15 min. For selective iNOS inhibitors, administration before the insult shows greater neuroprotective potential than post-insult treatment. The combination of nNOS and iNOS inhibition shows neuroprotective properties on histological, biochemical, and neurobehavioral outcome parameters when administered after the insult in a repeated dosing regimen. Thus, combined nNOS/iNOS inhibition with a repeated dosing regimen seems the most promising strategy to advance into human clinical trials. In fact, several phase II studies with 2-IB are currently underway, in addition to TH (NTR5221) as well as without TH in low-income countries (NCT01626924, EudraCT2015-003063-12).

Because of the wide variety in reported outcome measures, a clear-cut comparison between inhibitors based on outcome was difficult to make. Twelve studies report no neuroprotection on one or more outcome parameters after NOS inhibition. Potentially, this can be attributed to timing of the intervention or suboptimal dosing. When a NOS inhibitor is administered before the insult, the compound will be present in the tissues and circulation at the time of the actual insult, increasing the compound’s potential to exhibit neuroprotective effects. Most studies have tested one NOS inhibitor in a single dose. In studies testing different dosages, a higher dose often shows a better neuroprotective outcome, although some studies indicate a U-shaped effect. For 2-IB, the optimal dose in rats appears to be 30 mg/kg intraperitoneal (53). In piglets, increasing the dose by five times to 1.0 mg/kg intravenous does not provide greater neuroprotective properties compared with 0.2 mg/kg (57). Although most studies measured histological and biochemical outcome parameters associated with neuroprotection, the clinically most relevant parameter of improved neurobehavioral outcome was reported in four studies only. Yu et al. (45) and Ji et al. (49) showed that nNOS inhibition administered before the insult resulted in less deaths, and less neurobehavioral abnormalities in rabbits. Nijboer et al. (54) and Bjorkman et al. (57) report a (partial) neuroprotective effect for 2-IB on neurobehavioral outcome parameters in rats and piglets, respectively. Assessing neurobehavioral outcome requires a longer follow up period, which often involves intensive hands-on trained personnel especially in larger animal models, as well as validated tools to score the desired outcome parameter, making it very expensive. Using histological and biochemical markers provides researchers with a more time- and cost-effective alternative. Although data are limited, results on neurobehavioral outcome parameters, combined with results from histological and biochemical parameters, identify NOS inhibition as a potential neuroprotective strategy in humans.

Important differences exist between the adult and the neonatal brain with regard to susceptibility to injury, plasticity and cell death pathways. Therefore, adult animal models are not suitable to examine neuroprotective interventions for HIE. Across species, key brain maturation events regarding susceptibility and regenerative capacities have been identified at different moments before and after birth and are related to the developmental stage of the human neonatal brain (60–62). It is generally accepted that rats, at postnatal days 7–14 (P7–14), are comparable to near term/term human neonates with regards to cerebral cortex development (63, 64). The Vannucci–Rice model of unilateral common carotid artery ligation followed by a period of systemic hypoxia results in apoptotic-necrotic cell degeneration in P7–14 rats, similar to HIE (64–67). In term piglets aged 1–5 days, hypoxia leads to basal ganglia and somatosensory cortical injury, largely comparable to damage seen in human neonates after perinatal asphyxia (64, 68, 69). Introducing HI *in utero* to fetal rabbits provides animals with a motor phenotype similar to human cerebral palsy (64, 70). In term and preterm sheep models, hypoxia and asphyxia cause abnormalities in cerebral oxygen metabolism and hemodynamics as well as electrocortical brain activity comparable to human neonates after HI and basal ganglia injury representative for cerebral palsy (71–73).

Of interest is the potential role of sex-specific cell death pathways involved in HIE and possible sex-specific neuroprotective therapies. In general, females seem to be less susceptible to brain injury. This effect is seen across species, age groups, and origin of injury (74). In adult animal models, reduction in ischemic injury in females has been attributed to estradiol levels (74). Although estradiol will not be as predominant in prepubertal animal models, there is evidence of sexual dimorphism regarding sex steroids in central nervous system development in mice and rats (75, 76). Other studies show sex-specific cell death pathways leading to brain injury after HI both *in vitro* and *in vivo*. For instance, there is evidence that brain injury after HI in males is evoked by caspase-independent pathways whereas in females, caspase-dependent pathways are responsible (77–82). Therefore, neuroprotective agents such as NOS inhibitors that interact, either upstream or downstream, with the caspase-dependent pathway may be effective in females only.

The role of sex was only sparsely investigated in the studies included. For the majority of the studies (65%), the sex of the animals used was not reported. Six studies (23%) used rats of both sexes but have not reported sex-specific outcome. Yu et al. reported no outcome differences between sex for 7-NI and JI-9 but this statement was not supported by statistical analysis, possibly due to the small sample size in each of the groups (45). Nijboer et al. showed a statistically significant difference in histological and biochemical outcome parameters between sexes in rats, concluding that 2-IB was neuroprotective in female rats only (54). Other studies with different neuroprotective agents in both animals and humans also indicate a (potential) neuroprotective effect in females only (81–84).

Methodological quality assessment using the SYRCLE’s RoB tool resulted in only low and moderate scores for the publications used in this study. In all of the studies, at least on one or more items no information was available, forcing a score of 0 in that area. It is unknown whether these items were not adhered to during the experiment, or simply not included in the final manuscript due to regulations imposed by the editorial guidelines of the publishing journal. Unfortunately, it is not yet common practice to be as complete and precise in reporting data for animal studies as it is for human studies (33, 85). However, since this problem was addressed in a commentary published in the Lancet in 2002, awareness has been steadily increasing (86, 87). Fourteen of the studies included in this review were published in or before 2002; seven (50%) scoring low and an equal number scoring moderate. For the 12 included studies published in 2003 or later, 8 (67%) were awarded a moderate score. The SYRCLE’s RoB tool proved to be an adequate tool to consistently score the methodological quality of the included studies. However, this tool was developed recently and experience is still sparse. We would like to encourage future researchers to adhere to the items listed in this tool when conducting and reporting animal intervention studies to improve the methodological quality of studies as well as to use this tool when attempting a systematic review of animal literature. To illustrate the need for improvement in methodological quality and because of the possibility that low scores reflect lack of reporting and not lack of quality in the design of the study, we decided not to omit low quality studies nor did we emphasize the RoB scores when comparing the NOS inhibitors discussed in this study.

An important limitation of this study is that no independent statistics could be applied due to the large heterogeneity in study designs. Ideally, all NOS inhibitors should be tested in identical animal models with identical outcome measures. In reality, researchers over the past decades have used various animal models, dose and timing of NOS inhibitors, and reported outcome parameters. For the purpose of this review, we choose to report all of these and base our conclusions on the best available evidence. Based on this heterogeneity, these conclusions should be interpreted with caution.

Despite the low to moderate methodological quality according to the RoB tool, presented in Supplementary Material, and the lack of independent statistics, the evidence presented in this systematic review still indicates NOS inhibition as a promising strategy for (additional) neuroprotection in human neonates after perinatal asphyxia. Combined inhibition of nNOS and iNOS started as soon as possible after birth and in a repeated dosing regimen seems to have the best potential based on the combined outcome parameters, translation to clinical practice and methodological quality. Human studies (phase 2, open-label) with 2-IB, an inhibitor of both nNOS and iNOS, are currently taking place. Future clinical studies should make clear whether the sex-specific neuroprotective effect of drugs such as 2-IB observed in rats is present in humans as well. Furthermore, well designed placebo-controlled studies are needed to determine the safety of 2-IB in neonates and its effectiveness both with and without TH.

## Author Contributions

LF, AC, and FG were involved in study selection; LF, AC, and AH conducted the methodological quality assessment. All the authors discussed the results and read and approved the final version of the manuscript. LF drafted the manuscript; AC, AH, CN, CP-S, FB, TE, CR, and FG provided critical feedback to each draft.

## Conflict of Interest Statement

FB, FG, and CP-S are inventors of 2-iminobiotin as neuroprotective agent for neonates with HIE. CP-S is consultant for and shareholder of Neurophyxia BV’s-Hertogenbosch, The Netherlands. The other authors report no potential conflict of interest.
