# Supplementary material for: Nitric Oxide Synthase Inhibition as a Neuroprotective Strategy Following Hypoxic–Ischemic Encephalopathy: Evidence From Animal Studies
Source: Front Neurol. 2018 Apr 19;9:258. doi: 10.3389/fneur.2018.00258 (PMC5916957; doi:10.3389/fneur.2018.00258)
Supplement: Supplementary file 1 [file data_sheet_1.docx]

**Supplementary material**

Search string used for the EMBASE database. Slight modifications were made for the other databases if required.

*newborn OR fetus AND (piglet OR rat OR sheep OR mouse OR rabbit) AND ('brain hypoxia' OR asphyxia OR 'hypoxic ischemic encephalopathy' OR 'reperfusion injury':ti,ab OR 'fetus hypoxia' OR hypox*:ti,ab) AND ('nitric oxide synthase' OR 'nitric oxide synthesis' OR 'nitric oxide synthase inhibitor') NOT ('heart muscle ischemia'/exp OR 'heart muscle ischemia') NOT 'pulmonary hypertension' NOT 'heart' NOT 'necrotizing enterocolitis' NOT 'alveolar' NOT retina NOT kidney NOT 'preeclampsia' AND [english]/lim*

Table S1: Example of the RoB tool. For each study, each item was awarded 0, 1 or 2 points based on the available information in the manuscript. Item 3 was dropped for all studies.

|  | Type of bias | Description | Score  0/1/2 | Reason |
| --- | --- | --- | --- | --- |
| 1 | Selection bias - Sequence generation | Was the allocation sequence adequately generated and applied? |  |  |
| 2 | Selection bias - Baseline characteristics | Were the groups similar at baseline or were they adjusted for confounders in the analysis? |  |  |
| *3* | *Selection bias - Allocation concealment* | *Was the allocation adequately*  *concealed?* | *NA* | *Not scored* |
| 4 | Performance bias - Random housing | Were the animals randomly  housed during the experiment? |  |  |
| 5 | Performance bias - Blinding | Were the caregivers and/or  investigators blinded from  knowledge which intervention  each animal received during  the experiment? |  |  |
| 6 | Detection bias - Random outcome  assessment | Were animals selected at random  for outcome assessment? |  |  |
| 7 | Detection bias - Blinding | Was the outcome assessor  blinded? |  |  |
| 8 | Attrition bias - Incomplete outcome data | Were incomplete outcome  data adequately addressed? |  |  |
| 9 | Reporting bias - Selective outcome reporting | Are reports of the study free  of selective outcome  reporting? |  |  |
| 10 | Other -  Other sources of bias | Was the study apparently free  of other problems that could  result in high risk of bias? |  |  |
|  | Total |  | 0-18 |  |
